# ASDCD: Antifungal Synergistic Drug Combination Database

**DOI:** 10.1371/journal.pone.0086499

**Published:** 2014-01-24

**Authors:** Xing Chen, Biao Ren, Ming Chen, Ming-Xi Liu, Wei Ren, Quan-Xin Wang, Li-Xin Zhang, Gui-Ying Yan

**Affiliations:** 1 National Centre for Mathematics and Interdisciplinary Sciences, Chinese Academy of Sciences, Beijing, P. R. China; 2 Academy of Mathematics and Systems Science, Chinese Academy of Sciences, Beijing, P. R. China; 3 University of Chinese Academy of Sciences, Beijing, P. R. China; 4 South China Sea Institute of Oceanology, Chinese Academy of Sciences, Guangzhou, P. R. China; 5 Chinese Academy of Sciences Key Laboratory of Pathogenic Microbiology and Immunology, Institute of Microbiology, Chinese Academy of Sciences, Beijing, P. R. China; 6 School of Life Science, University of Science and Technology of China, Hefei, P.R. China; Institute of Microbiology, Switzerland

## Abstract

Finding effective drugs to treat fungal infections has important clinical significance based on high mortality rates, especially in an immunodeficient population. Traditional antifungal drugs with single targets have been reported to cause serious side effects and drug resistance. Nowadays, however, drug combinations, particularly with respect to synergistic interaction, have attracted the attention of researchers. In fact, synergistic drug combinations could simultaneously affect multiple subpopulations, targets, and diseases. Therefore, a strategy that employs synergistic antifungal drug combinations could eliminate the limitations noted above and offer the opportunity to explore this emerging bioactive chemical space. However, it is first necessary to build a powerful database in order to facilitate the analysis of drug combinations. To address this gap in our knowledge, we have built the first Antifungal Synergistic Drug Combination Database (ASDCD), including previously published synergistic antifungal drug combinations, chemical structures, targets, target-related signaling pathways, indications, and other pertinent data. Its current version includes 210 antifungal synergistic drug combinations and 1225 drug-target interactions, involving 105 individual drugs from more than 12,000 references. ASDCD is freely available at http://ASDCD.amss.ac.cn.

## Introduction

Researchers across many scientific domains have responded to the increasing lack of effective drugs and equally increasing drug-resistant pathogenic strains by studying the feasibility of combinatorial therapy. Drug combinations could simultaneously affect multiple subpopulations, targets, and diseases [1]–[4], and they are more efficacious than single drugs aimed at single targets [2], [5]. Also, the development of drug resistance can be slowed down by the use of drug combinations because biological systems are less able to compensate for the simultaneous action of two or more drugs [1], [2], [4]–[7]. At present, drug combinations have been classified into three types: additive, synergistic, and antagonistic [8]–[10]. Synergistic interaction requires fewer drugs to produce a given effect than their single use [11], thus increasing therapeutic efficacy and reducing toxicity and side effects [1], [12]. Several models are helpful in understanding synergistic effect: 1) the physical interaction model suggests that two drugs with physical interactions tend to be a more potential synergistic combination [13];2) the same target model involves two synergistic drugs that tend to target different sites of the same protein [14]; 3) the parallel pathway inhibition model proposes that two drugs will be synergistic if they inhibit two proteins on parallel pathways essential for an observed phenotype [15]; and, finally, 4) the bioavailability model suggests that two drugs will be synergistic if one drug’s action helps another drug’s availability in the target cells [4].

The history of drug combinations can be traced back 1900 years to Huangdi Neijing. Nowadays, drug combinations have been widely used in biomedical research and clinical practice to treat disease [1], [16]–[25]. Traditional Chinese Medicines (TCM) and well-established treatments for AIDS, cancer, and infectious diseases are vivid examples [1], [5]. TCM combines different compounds to increase therapeutic effectiveness, while minimizing toxicity and side effects [26]–[29]. A combination of at least three active antiretroviral medications known as the AIDS cocktail not only delays the progression of AIDS, helps rebuild and maintain the immune system, and reduces complications, but also helps prevent drug resistance. Current cancer treatment also relies heavily on such drug combinations as anthracyclines, platinum drugs, and taxanes [17]–[24], [30]–[32]. These impressive successful examples fully illustrate the benefits of drug combinations.

With increasing mortality rates of immunocompromised patients affected by invasive fungal infections and emergent drug resistance, new therapeutic strategies and effective antifungal drugs with new mechanisms of action are urgently needed. Thus, in-depth analyses of known successful and unsuccessful drug combinations would yield a greater understanding of the patterns of synergistic drug combinations and, at the same time, accelerate the development of new drug combinations [33]. Synergistic drug combinations are a promising strategy and tend to improve therapeutically relevant selectivity [34]. Through systematic screening of two-component combinations, fungistatic and analgesic agents can show synergistic activity against drug-resistant *Candida albicans* without significant cytotoxicity. Borisy et al., for example, found 22 hits from 30 different dual-drug combinations, six of which are comprised of antifungal and nonantifungal agents, while the other 16 pairwise combinations are comprised of two nonantifungal agents [3]. Natural products have played an important role in antifungal drug discovery since the first approved “griseofulvin” isolated from *Penicillium griseofulvum*, followed by the polyene antibiotics and echinocandin antifungals [35]. Previously in our lab, we constructed a microbial natural product library, and using the High-Throughput Synergistic Screening (HTSS) approach, compounds such as beauvericin, berberine, cyclosporin A, geldanamycin, lovastatin and radicicol could gain synergistic effect with azoles to inhibit different fungal pathogens [36]. For example, the combination of beauvericin and ketoconazole presented a significant synergistic anti-*Candida* effect. This combination prolonged the lifespan of fungi-infected mice and significantly reduced the toxicity of ketoconazole when administered in high doses to human cells [36]. High-throughput synergy screening is an effective strategy to discover new drug combinations, but it is expensive and time-consuming. Therefore, systematic biology and computational predictive methods are urgently needed to discover potential antifungal drug combinations. To implement such methods, it is necessary to collect known experimentally verified synergistic antifungal drug combinations as training samples. Up to now, however, no specialized synergistic antifungal drug database has been available for mathematical and computational research.

To address this gap in our knowledge, we built the first Antifungal Synergistic Drug Combination Database (ASDCD) as a repository for the growing antifungal data and as a means of tracking scientific developments in this area. ASDCD would be specially designed for the input of synergistic antifungal drug combinations and provide a powerful search tool and research platform to facilitate drug combination analysis and new antifungal drug development. ASDCD includes information about published synergistic antifungal drug combinations, chemical structures, drug targets, target-related signaling pathways, drug indications and other pertinent data. Its current version includes 210 antifungal synergistic drug combinations and 1225 drug-target interactions involving 105 individual drugs.

## Data Collection

**Figure 1:**
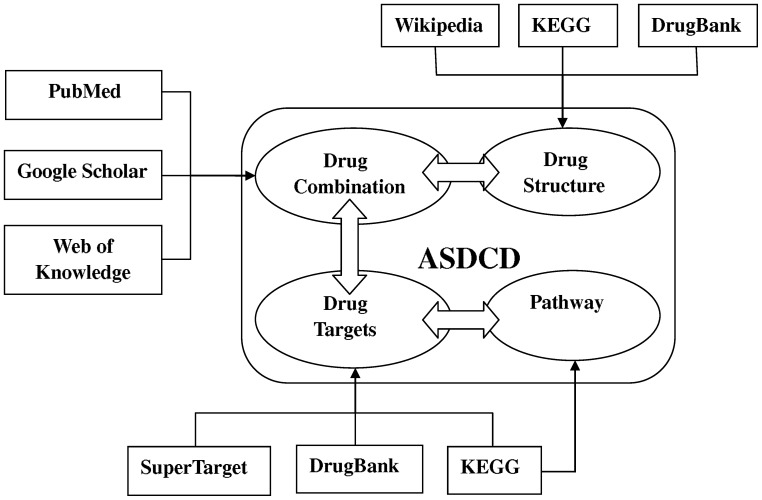
Schematic illustration of ASDCD. Antifungal drug combinations were collected from PubMed, Google Scholar and Web of Knowledge. Information about chemical structure, drug-target interactions, and target-related pathways was primarily collected from KEGG, DrugBank and SuperTarget.

To obtain high-quality synergistic antifungal drug combinations, we used PubMed, Google Scholar and Web of Knowledge and employed the keyword ‘synergy’ to collect experimentally identified combinations from more than 12,000 articles published before June 2011. We also searched articles with the keywords ‘synergic’, ‘synergistic’, ‘synergism’, ‘interaction’ and ‘combination’ to avoid missing data. Drug chemical structures were collected from DrugBank [37], KEGG [38] and Wikipedia. Drug targets were retrieved from KEGG, DrugBank and SuperTarget [39], while the target-related signaling pathways mainly came from KEGG. The overall collection process is shown in Figure 1.

## Database Content and Web Interface

### Homepage

**Figure 2:**
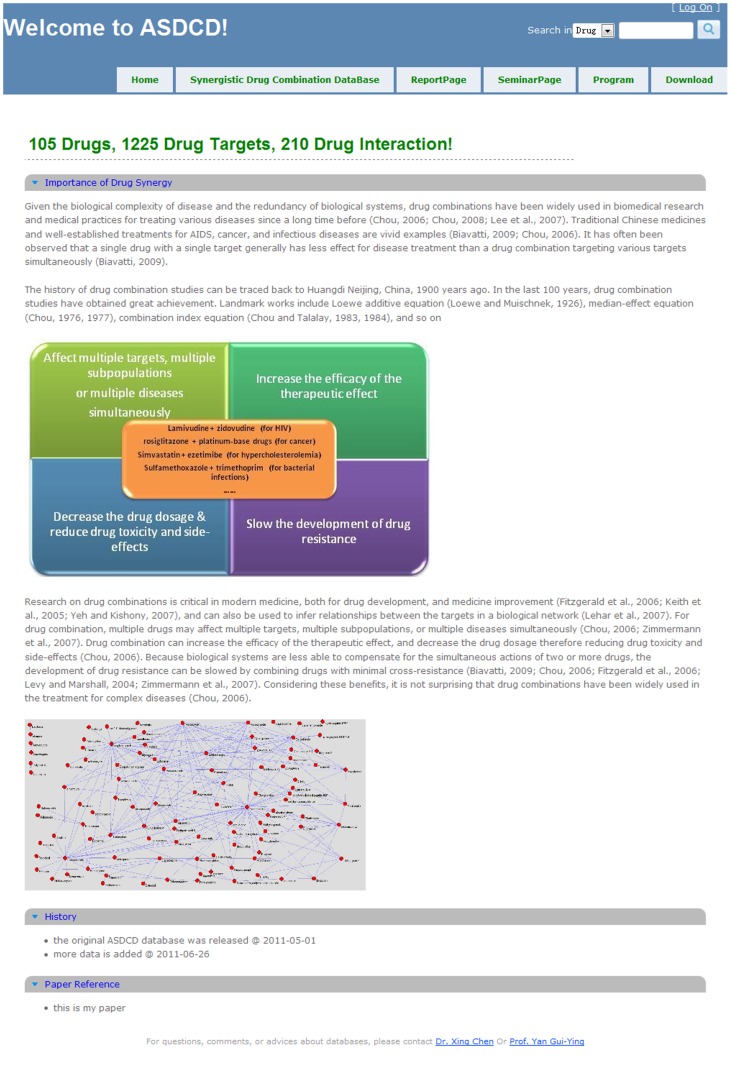
Screenshot of ASDCD web interface is shown. The number of individual drugs, drug target interactions, and synergistic drug combination, relevant history and the significance of synergistic drug combination, some basic information about ASDCD and an easy-to-use search tool are provided.

At present, ASDCD can be freely accessed at http://ASDCD.amss.ac.cn. The web interface is designed to be very user-friendly, enabling researchers to easily navigate the hyperlinks to retrieve data (Figure 2). At the top of the homepage, users will find the pertinent statistics, including the number of individual drugs, drug-target interactions, and synergistic drug combinations in the database. The page goes on to present the relevant history and significance of synergistic drug combinations, including a figure which fully demonstrates the benefits of combinatorial drug therapy and some well-established drug combinations used for cancer, HIV, bacterial infections and others. Then, synergistic drug combination network based on the datasets in this database was shown in the homepage. Finally, some basic information about ASDCD is provided, such as version and release data. For fast retrieval of information, a search tool is found at the top right corner of the page, allowing a fast and convenient way to search for drugs and targets. When drug keywords are input, all possible drugs containing the keywords are listed. Similarly, when target keywords are input, all targets including keywords and drugs interacting with them are listed.

### Drug Combination Page

When the hyperlink ‘Synergistic Drug Combination DataBase’ is selected, all drugs having combinations in ASDCD are listed. On this page, users will find two rows of uppercase letters, A to Z, and numbers from 0 to 9. The upper row is the index for drugs, and the lower row is the index for targets. Thus, by selecting either a letter or number in the upper row, all drugs with names beginning with this letter or number are listed. In the lower row for targets, the search strategy is similar.

Below these two rows of letters and numbers, all the drugs in ASDCD are listed. Drugs are numbered and ranked in alphabetical order. For each drug, basic information can be seen, such as drug ID, drug name, and chemical structure. For each drug, ASDCD also offers three convenient links for researchers: drug target, target-related signaling pathway, and its synergistic interactions in combination with other drugs. If users are interested in detailed information about a certain drug, it is only necessary to select drug name, and all the available information about it, including basic information, drug interaction, drug target, and target-related pathway, is shown. Basic information consists of drug ID, name, detail, indications and chemical structure. Drug interactions include all other drugs with which the highlighted drug might have synergistic effect. The user will also find related references, including title, author, year, journal, and PMID link, that have reported on the interactions of this particular synergistic drug combination or its implementation, using both *in vitro* and *in vivo* experiments. A listing of drug targets for the highlighted drug is also provided, together with its corresponding reference links. Through these links, users can search for information about the interactions between drug and target based on the literature and such drug databases as KEGG, DrugBank and SuperTarget [39]. The detailed information for any specific target can be easily found in these databases. In addition, ASDCD offers target-related pathway information for each drug. Cross referencing of drugs, targets and pathways helps researchers to investigate the mechanisms of action and efficacy of many drug combinations, thus aiding in the development of new drugs.

### Comment Function

ASDCD provides a comment function that allows other researchers to comment on the data in the database, provide important synergistic drug combinations that are not documented, or make suggestions. Once verified by the webmaster, the submitted drug combination will be included in the database and made available at once.

## Compared with DCDB

DCDB (Drug Combination Database), which was constructed by researchers in Zhejiang University, [33] is the first database devoted specially to the R&D of multicomponent drugs. Its current version collected 499 drug combinations, involving 485 individual drugs. The drug combinations in DCDB were manually collected from PubMed and the U.S. Food and Drug Administration (FDA) Orange-Book.

Unlike DCDB, which collects many kinds of drug combinations for a variety of human diseases, ASDCD focus on synergistic drug combinations for the therapy of fungal infection. Fungal infection is a very serious infectious disease. Combination therapy has been recognized as an important and effective way for treating fungal infection since 30 years ago [40], when Bennett et al compared amphotericin B (AmB) alone and in combination with 5-fluorocytosine (5FC) in the treatment of cryptococcal meningitis [40], [41]. Undoubtedly, ASDCD could be a very useful and convenient resource of drug combination for researchers and doctors during the process of research and clinical therapy.

**Figure 3:**
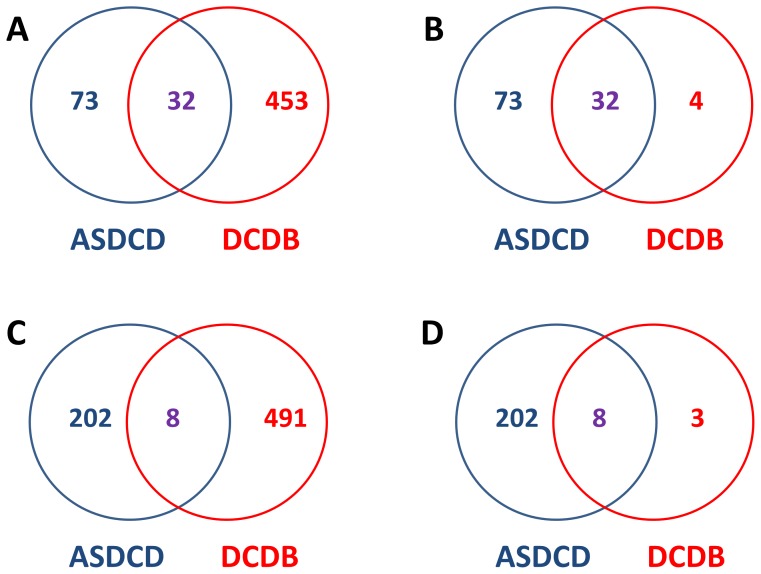
ASDCD is compared with another drug combination database DCDB. A) the comparison between ASDCD and DCDB in the term of the number of drugs in the database; B) the comparison in the term of the number of antifungal drugs; C) the comparison in the term of the number of drug combinations; D) the comparison in the term of the number of antifungal drug combinations. From above comparison, it can be easily observed that few antifungal drugs and drug combinations were collected in DCDB and ASDCD has provided much more drugs and drug combinations for the treatment of fungal infection.

Although there are also antifungal drug combinations in DCDB, the number of individual antifungal drugs and drug combinations is far from enough, compared with the ASDCD database. Especially, there are 105 antifungal drugs in ASDCD, but you can only find 36 antifungal drugs in DCDB (See Figure 3). What’s more, Fluconazole and Caspofungin, two kinds of very commonly used antifungal drug in clinical therapy, have not been collected by DCDB. As for the number of drug combinations, there are 210 antifungal drug combinations in ASDCD, but only 11 antifungal drug combinations were collected in DCDB (See Figure 3). Besides, in contrast to DCDB, information about drug combination and drug target in ASDCD is the newest and updated frequently.

Compared with DCDB, ASDCD indeed has some little shortages. On the one hand, ASDCD only provides drug combinations of two individual drugs, not considering the combination of three or more drugs. On the other hand, some more detail information about individual drugs, drug targets and drug combinations, like Brand Name, ATC Code, and Mechanism of Action, has not been provided by ASDCD. In future, we would improve our database by introducing high-order antifungal drug combinations and more detailed information about drugs, drugs combinations and drug targets.

## Fluconazole Synergistic Combination Network

Fluconazole, one of the most commonly used triazole antifungal drugs, is commonly marketed under the trade name Diflucan or Trican (Pfizer) (http://en.wikipedia.org/wiki/Fluconazole). It is often used in the treatment and prevention of superficial and systemic fungal infections, such as yeast infections of the mouth, throat, esophagus, abdomen, lungs, blood, and other organs (http://www.nlm.nih.gov/medlineplus/druginfo/meds/a690002.html). It is also introduced to treat meningitis caused by fungus.

**Figure 4:**
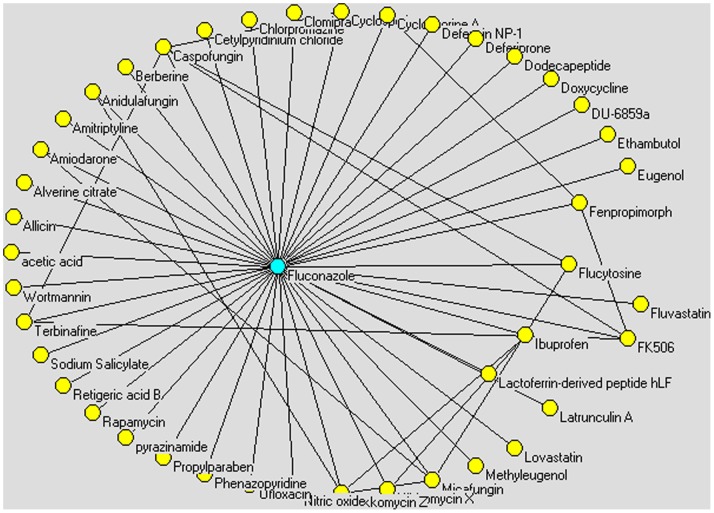
Fluconazole synergistic combination network includes fluconazole and other drugs yielding synergistic effect with fluconazole as nodes and the synergistic interactions between all the drugs in the network as edges. In ASDCD, fluconazole obtains synergistic effect with as many as 42 drugs for antifungal treatment.

Moreover, fluconazole can be used to prevent yeast infections in patients who are likely to become infected because they are being treated with chemotherapy or radiation therapy before a bone marrow transplant (http://www.nlm.nih.gov/medlineplus/druginfo/meds/a690002.html). Generally speaking, it is a drug indicated for the treatment and prophylaxis of fungal infections where other antifungal drugs have failed or are not tolerated, such as candidiasis, onychomycosis, cryptococcal meningitis, tinea corporis or tinea cruris. It is used as a first-line drug for the following indications: coccidioidomycosis, cryptococcosis, histoplasmosis, and prophylaxis of candidiasis in immunocompromised people [42]. In ASDCD, it will be found that fluconazole obtains synergistic effect with as many as 42 drugs for antifungal treatment. For example, fluconazole achieves synergistic effects with berberine, even in drug-resistant *Candida albicans* infections [43]. It has been reported that fluconazole combined with berberine can increase mitochondrial membrane potential, decrease intracellular ATP level, inhibit ATP-synthesis activity, and increase the generation of endogenous reactive oxygen species (ROS) in fluconazole-resistant strains, thereby inhibiting the drug-resistant *C. albicans* strain. The fluconazole synergistic combination network is shown in Figure 4, including fluconazole and other drugs yielding synergistic effect with fluconazole as nodes and the synergistic interactions between all the drugs in the network as edges.

## Conclusions and Future Prospects

ASDCD is the first drug combination database devoted to antifungal drug research. Its current version includes 210 antifungal drug combinations, involving 105 individual drugs. Considering the ever-growing demand and interests from both academia and commercial sectors, ASDCD will be updated at least twice a year. In the future, we will incorporate additional web analysis and predictive tools to further promote drug combination research and improve antifungal drug development.
